# A Novel Multi-Gene Detection Platform for the Analysis of miRNA Expression

**DOI:** 10.1038/s41598-018-29146-7

**Published:** 2018-07-16

**Authors:** Chia-Hsun Hsieh, Wei-Ming Chen, Yi-Shan Hsieh, Ya-Chun Fan, Pok Eric Yang, Shih-Ting Kang, Chun-Ta Liao

**Affiliations:** 1 Division of Hematology-Oncology, Department of Internal Medicine, Chang Gung Memorial Hospital, Taoyuan, Taiwan; 2 Department of Application Development, Quark Biosciences, Inc., Zhubei City, Hsinchu, Taiwan; 3 Department of Otorhinolaryngology, Head & Neck Surgery, Linkou Chang Gung Memorial Hospital and Chang Gung University, Taoyuan, Taiwan

## Abstract

The study of miRNAs and their roles as non-invasive biomarkers has been intensely conducted in cancer diseases over the past decade. Various platforms, ranging from conventional qPCRs to Next Generation Sequencers (NGS), have been widely used to analyze miRNA expression. Here we introduced a novel platform, PanelChip™ Analysis System, which provides a sensitive solution for the analysis of miRNA levels in blood. After conducting miRQC analysis, the system’s analytical performance compared favorably against similar nanoscale qPCR-based array technologies. Because PanelChip™ requires only a minimal amount of miRNA for analysis, we used it to screen for potential diagnostic biomarkers in the plasma of patients with oral cavity squamous cell carcinoma (OSCC). Combining the platform with a machine learning algorithm, we were able to discover miRNA expression patterns capable of separating healthy subjects from patients with OSCC.

## Introduction

MicroRNAs (miRNAs) are 18–25 nucleotides long highly conserved, non-coding RNAs involved in gene regulation^1,2^. Since the discovery of the first miRNA in *C. elegan* in 1993, many more miRNAs have been identified to exist in humans and other multicellular organisms^1,3–6^. The human genome comprises of more than 2500 miRNAs, some of which are implicated in many diseases, including cancer^7–11^. The first link between cancer and miRNAs was discovered in 2002^12^. Two miRNA genes were identified to be deleted in 50% of chronic lymphocytic leukemia (CLL) patients, suggesting that these two miRNAs were key factors involved in the causation of CLL^12^. Since then, a plethora of reports has shown that many miRNAs, acting as oncogenes and/or tumor suppressor genes, play critical roles in the pathophysiology of a significant number of human cancers^9,11,13–25^. The precise molecular mechanisms of the regulation of gene expression by miRNAs in tumor cells remain to be elucidated. Nonetheless, the study of the expression patterns of individual miRNAs in various cancers has yielded large sets of data that would be of important diagnostic and prognostic values^9,11,17,26–30^.

More recently, miRNAs have gained tremendous traction as a novel class of promising cancer biomarkers. In addition to their unique expression pattern in various cancer types, cell-free miRNAs are remarkably stable in blood, making them invaluable in clinical settings as blood samples can be collected rather easily from individuals^30–33^. Various types of platforms, from conventional qPCRs, high-density qPCR-based array technologies, hybridization systems to NGS, have been used to discover and validate miRNA biomarkers by expression analysis. Conventional qPCRs are often used in the validation of biomarkers, while hybridization methods such as microarrays and/or NGS are being used at the discovery phase. In this manuscript, we introduced a novel multi-gene expression profiling system, PanelChip™ Analysis System, capable of concurrently analyzing the expression of 96 miRNA biomarkers per assay^34^. PanelChip™, a key component of the system, is a nanowell chip preloaded with primers and probes amplifying the intended RNA targets to be analyzed^34^. Another key component of the system is PanelStation™, the thermal cycler carrying out qPCR reactions on PanelChip™ (Both components have been granted U.S. patents: US9168533^35^ and US9724692^36^). We’ve developed two PanelChip™ assays to analyze miRNA biomarkers, and demonstrated in this paper that the system performed favorably against other platforms in a number of miRQC analytical tests^37^. With qPCR as the underlying detection technology, the system is well-suited for the measurement of miRNA levels in body fluids, with a required starting material amount of as little as 0.2 ng total miRNAs.

To illustrate its capability in analyzing miRNA expression in body fluids, we’ve also utilized the system and machine learning algorithm for the discovery of miRNA biomarkers in patients with OSCC. Though there are many clinicopathological risk factors associated with OSCC, no accepted molecular biomarkers have been identified yet^38,39^. We were able to discover miRNA expression patterns capable of differentiating healthy subjects from patients with OSCC. Therefore, the combination of a medium density platform, such as PanelChip™ Analysis System, and deep learning analysis provides a high-powered solution for fast biomarker discovery and validation.

## Results

### Evaluation of the Performance of miRNA Expression Profiling using PanelChip™ Analysis System

**Figure 1:**
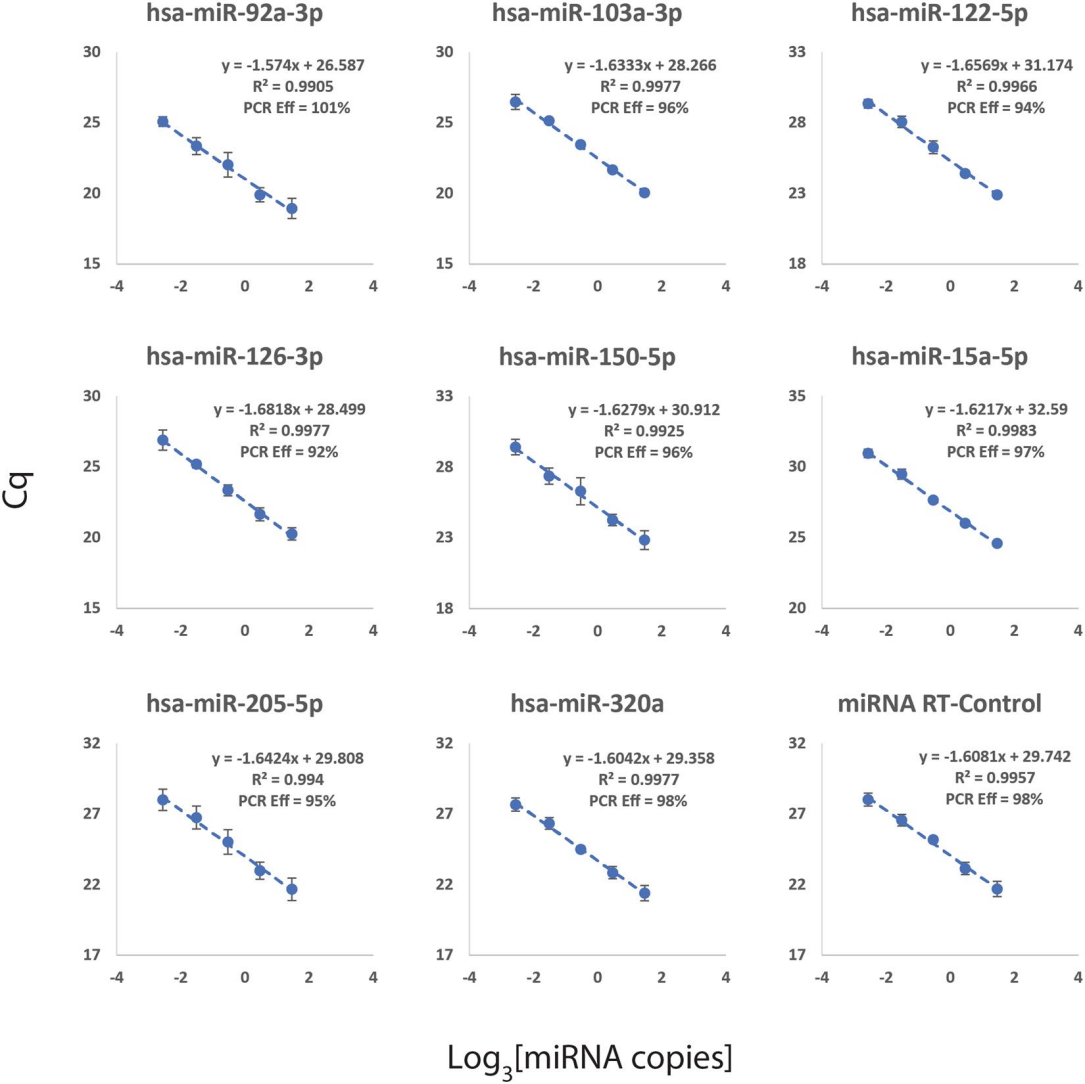
PCR efficiency of 9 representative miRNA assays on miRSCan™ PanCancer Chips. Real-time qPCR was carried out for each miRNA cluster using 3-fold serially diluted cDNA template synthesized from Universal Human miRNA Reference RNA (UHmiRR; miRQC A). The resulting Cq values were plotted against the respective miRNA concentrations to derive the PCR efficiency for each assay. All 9 assays fell within acceptable PCR efficiency of 90–110%.

PanelChip™ Analysis System, a novel multi-gene expression profiling technology, was first evaluated for its performance in analyzing miRNA expression^34^. Two PanelChips™, miRSCan™ PanCancer Chips 1 & 2 (material and methods) were designed and developed to study the expression of miRNAs in cancer diseases. The miRSCan™ Chips analyze a total of 164 potential miRNA biomarkers, which were selected from miDatabase™ (Yang, K.C. *et al*. in submission). Biomarkers were selected based on a number of criteria, but mainly for their implications in cancer diseases and presence in human plasma. The primers used to detect miRNA signals were designed using miPrimers™ methodology^40^. We first determined the qPCR efficiency of the miRNA assays, or clusters, on miRSCan™ PanCancer Chips to demonstrate that they were in an acceptable range. As illustrated in Fig. 1, the qPCR efficiency for 9 representative miRNA clusters all lay within the acceptable range of 90–110%^41^. We have also included the qPCR efficiency of other miRNA clusters in Supplementary Table S1.

Since miRSCan™ PanCancer Chip/PanelChip™ Analysis System is a novel solution for analyzing miRNA expression patterns, we next assessed its analytical performances using miRQC analysis methods developed by Mestdagh *et al*. for evaluating miRNA measurement platforms^37^. We evaluated the titration response, reproducibility, accuracy of expression differences, detection sensitivity and specificity using various standard samples based on the miRQC study (see Supplementary Table S2).

### Titration response

To evaluate titration response, Universal Human miRNA Reference RNA (UHmiRR; miRQC A) and Human Brain RNA (HBR; miRQC B) were first mixed to create 4 samples of various titrations (100% of UHmiRR: A, 100% HBR: B, 75% UHmiRR + 25% HBR: C, 25% UHmiRR + 75% HBR: D). If miRNA x has a higher expression in sample A than B (Ax > Bx), then Ax > Cx > Dx > Bx^37^. Conversely, if miRNA x has a higher expression in sample B than A (Bx > Ax), then Bx > Dx > Cx > Ax^37^. The ability to correctly determine the order is defined as titration response. We analyzed the titration response of 164 miRNAs on miRSCan™ PanCancer Chips 1 & 2 using the analysis method from Mestdagh *et al*. to determine the AUC value (see Table 1). Titration response was quantified by calculating the area between the theoretical cumulative distribution, and the cumulative distribution of PanelChip™ Analysis System. High titration response represents the platform’s capability to detect small expression changes. The AUC titration response of PanelChip™ was 0.75, a decent number for nano-volume qPCR-based technology.

**Table 1:** Summary of miRQC performance parameters of PanelChip™ Analysis System.

Experiment	Parameter	Value
PanelChip™ Analysis System	Clusters (Total of 164 before cutoff)	138
	Cutoff	Cq < 34
Reproducibility	Unique double positives (%)	94.93
	Fraction single positives (%)	5.07
	Expression range(log2-units)	18.64
	ALC	0.58
Titration	AUC titration response	0.75
Specificity	Off-target combinations with cross reactivity (%)	0
	Median relative cross-reactivity (%)	0
No-template control	Positive miRNAs	16
Plasma miRNAs	Detected miRNAs	78

PanelChip™ Analysis System was evaluated on parameters including reproducibility, titration, specificity, no-template control, detection of plasma miRNAs and differential expression.

Clusters, total number of miRNA assays on miRSCan™ PanCancer Chip 1&2.

Cutoff, miRNA assays with Cq value of more than 34 were removed.

Reproducibility, the ability to detect the same number of miRNAs in two replicates.

Unique double positives, % of miRNAs detected in replicates.

Fraction single positives, % of miRNAs detected in one of the two replicates.

Expression range, detectable expression range of unique double positives.

ALC, area left of cumulative distribution curve where lower ALC is indicative of higher reproducibility. Titration, the ability to correctly predict the order of miRQC A, B, C, and D based on miRNA expression.

AUC, area under the curve which is a single scale-invariant measure of platform titration response. Specificity, the specificity of miRNA primers.

No-template control, MS2 Phage RNA only.

Plasma miRNAs, total number of miRNAs detectable by the platform.

### Reproducibility

**Figure 2:**
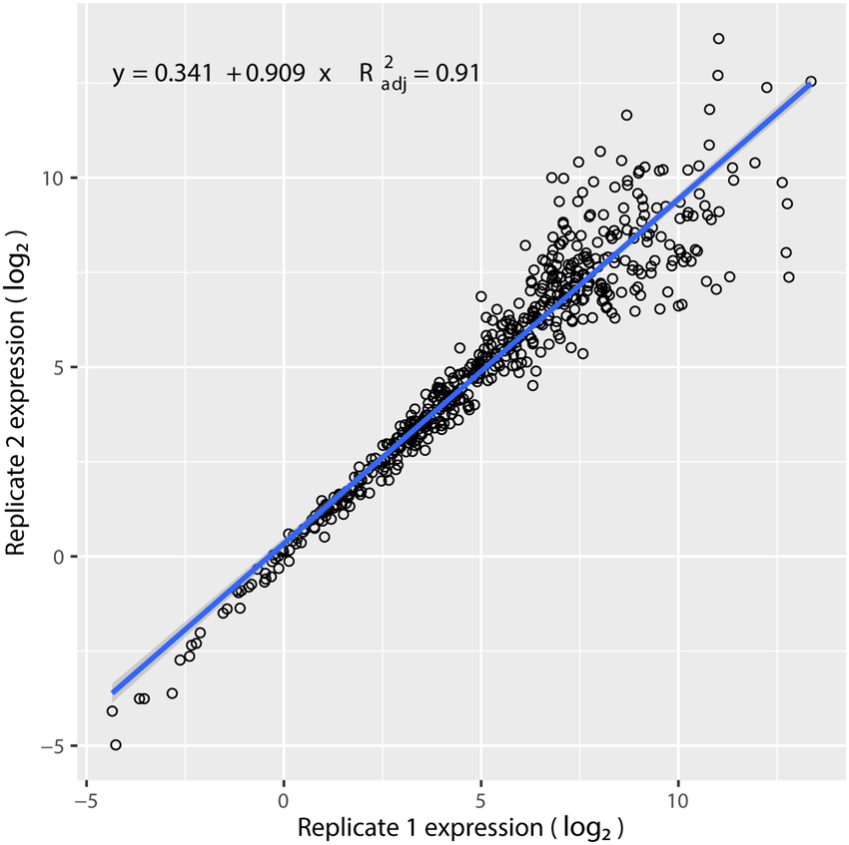
Correlation between miRQC sample replicates. Real-time qPCR was carried out on duplicate miRQC samples to evaluate the reproducibility between miRSCan™ PanCancer chips. Correlation analysis was performed using the resulting two qPCR datasets of duplicate samples (samples 1, 3, 5, 7 for replicate 1 and samples 2, 4, 6, 8 for replicate 2). Filled circles represented data from replicate 1, and open circles represented data from replicate 2. Correlation of 0.91 indicates that miRSCan™ PanCancer chips have high reproducibility.

**Figure 3:**
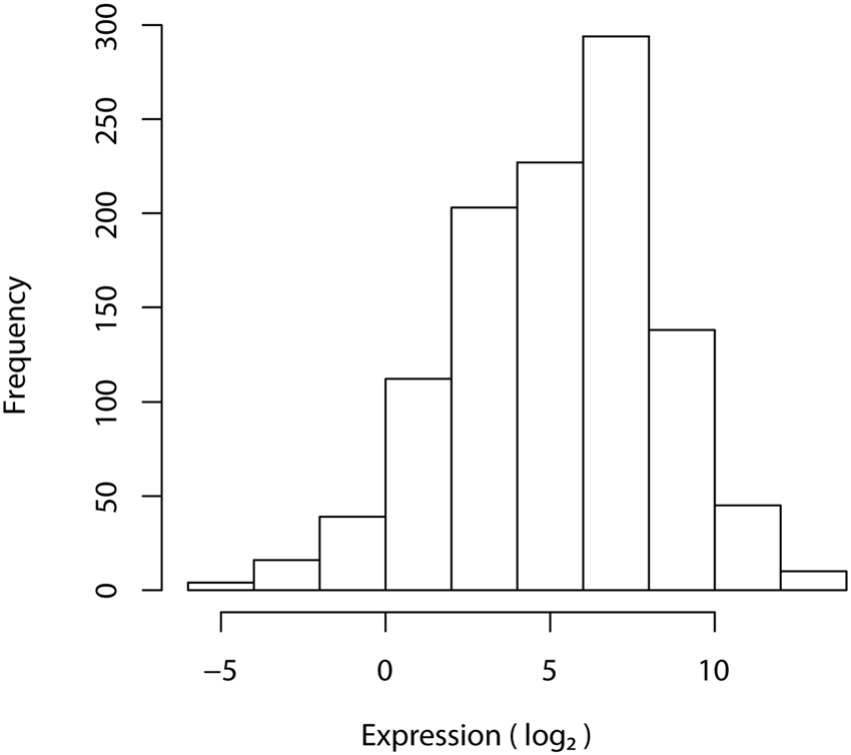
Expression distribution of detectable miRNAs from double positive replicates. Real-time qPCR was carried out on duplicate miRQC samples. Double positives are miRNAs detected in both duplicates. 131 double positives out of 164 total miRNA candidates were detected after applying the detection cut-off (Cq < 34). Expression range of the double positives is 18.64 log2-units, showing the system’s ability to detect a wide range of template concentrations.

Duplicated miRQC samples (see Supplementary Table S2, samples 1–8, miRQC A–D) were analyzed to evaluate the reproducibility of the PanelChip™ Analysis System. Correlation of expression comparing miRQC A–D replicates revealed two populations: (1) double positives, where the expression of the miRNAs was determined in both replicates and (2) single positives, where the expression of the miRNAs was determined in only one of the replicates^37^. Figure 2 depicted a strong correlation of 0.91 between 2 sets of miRQC samples, which showed high reproducibility of the system. Utilizing the detected expression values of double positives, we also determined the expression range of the system to be 18.64 log2-units, illustrating the system’s ability to detect a wide range of template concentrations (Fig. 3).

### Detection rate and sensitivity

Detection sensitivity was first evaluated based on the number of double positives that were detected in the replicates^37^. PanelChip™ Analysis System detected 94.93% double positives, equivalent to 131 out of the 138 miRNAs that remained after cut-off. One of the strengths of the system is its capability to detect very low amount of circulating miRNAs (see Table 1). Low amounts of miRNA, ~0.2 ng, were used to study the expression patterns of healthy donors (HD) and patients (see clinical study below). Using plasma as samples (miRQC samples 12–15), we detected 78 circulating miRNAs, illustrating that PanelChip™ has a very sensitive detection rate (>48%) compared to other existing qPCR-based platforms. Furthermore, compared to multi-gene qPCR platforms such as OpenArray®, pre-amplification is required for PanelChip™ Analysis System.

### Specificity

System and assay specificity was evaluated using MS2-phage RNA without any miRNAs^37^. The system/assay showed a 10% positive signal in no-template control reactions, which was in line with the range of 6.79% to 10.79% observed in other existing small-volume qPCR platforms^37^. To further evaluate specificity, 3 synthetic miRNAs of let-7 family (let-7a-5p, let-7b-5p, and let-7c-5p) were spiked into MS2-phage RNA. The sequences of the three let-7 family members only differed by one or two nucleotides. After cDNA synthesis and amplification, PanelChip™ Analysis System showed excellent specificity with no cross reactivity for at least 3 let-7 family members (Table 2)^40^.

**Table 2:** Percentage of cluster cross-reactivity of let-7 miRNA family members.

miRNA	hsa-let-7a-5p	hsa-let-7b-5p	hsa-let-7c-5p
hsa-let-7a-5p	100	0	0
hsa-let-7b-5p	0	100	0
hsa-let-7c-5p	0	0	100
			Cross Reactivity: 0

### Dynamic range

**Figure 4:**
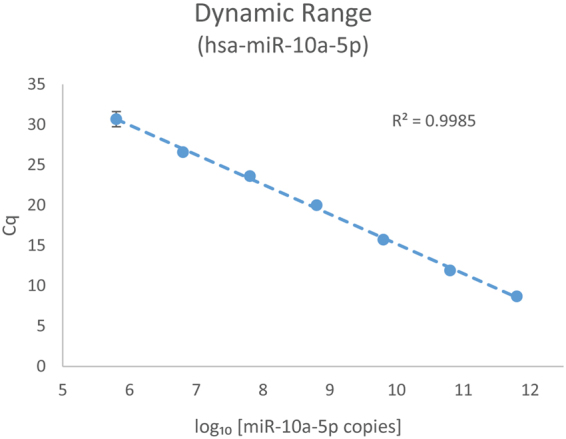
Dynamic range of known concentration of spike-in synthetic has-miR-10a-5p. A set of 10-fold serial dilutions of synthetic hsa-miR-10a-5p miRNA were spiked into the same amount of Universal Human miRNA reference RNA (samples 16–22, miRQC A) to generate cDNA for qPCR analysis. 20 ng of the RNA input was used for the spike-in test. qPCR results illustrated a dynamic range of at least 7 orders of magnitude ranging from 80 to 8 × 10^7^ copies per nanowell.

To determine the dynamic range of detection, synthetic miR-10a-5p was serially diluted into 20 ng of Universal Human miRNA Reference RNA (UHmiRR; miRQC A); the reference miRNA contains a negligible amount of endogenous miR-10a-5p^37^. The results showed that the system was capable of detecting the spike-in miRNA for at least 7 orders of magnitude, ranging from 80 to 8 × 10^7^ copies per nanowell (Fig. 4). The dynamic range again demonstrated the system’s sensitivity to detect miRNAs from a wide range of template concentrations.

All these results showed that PanelChip™ Analysis System performed well in the miRQC study. Ultimately, the goal of RNA expression profiling platforms is to determine differential expression between sample groups. To illustrate this, we next used PanelChip™ Analysis System and miRSCan™ PanCancer Chips 1 & 2 to compare the plasma miRNA expression pattern between HD and patients with OSCC.

### Evaluation of plasma miRNA levels in patients with OSCC and healthy subjects using PanelChip™ Platform and Support Vector Machine

To determine if we can discover plasma miRNA expression pattern specific to patients with OSCC using PanelChip™ Analysis System, 38 patients with OSCC and 84 HD were enrolled for the study. The basic demographic and clinical information of each patient are listed in Supplementary Table S3. Small RNAs were isolated from the plasma samples and synthesized into cDNA for analysis on miRSCan™ PanCancer Chips 1 & 2. Since support vector machine (SVM) has been used in several studies to develop cancer/disease classifiers, we decided to utilize it to build an OSCC classifier based on the resulting miRNA expression^42–44^. The given dataset, containing two classes obtained from patients with OSCC and HD, was divided into a training set (90% of the dataset) and a testing set (10% of the dataset). Based on the 10-fold cross-validation, the training set was used to train OSCC classifier and the testing set was used to evaluate the performance of OSCC classifier. From our analysis, OSCC classifier produces a sensitivity of 81.6% and a specificity of 98.8% for oral cavity cancer prediction (Table 3). Since there are no molecular biomarkers for the screening of OSCC, our classifier can be potentially valuable for the diagnosis of oral cavity cancer. The classifier is also suitable for identifying individuals who are healthy because of its high specificity. For the purpose of this study, the diagnostic performance of OSCC classifier did not take into account factors such as clinicopathological state of the tumor and the patients’ age, sex, ethnicity or betel nut chewing/smoking habits.

**Table 3:** Confusion matrix for classification of OSCC classifier.

	No. of Patients	True Class	
		OSCC	HD
Predicted Class	OSCC	31 (TP)	1 (FP)
	HD	7 (FN)	83 (TN)

HD, healthy donors.

OSCC, patients with OSCC.

True Class, the actual clinical status of the subjects.

Predicted Class, the predicted clinical status of the subjects using the algorithm.

TP, true positives.

FP, false positives.

FN, false negatives.

TN, true negatives.

Sensitivity, TP/(TP + FN) = 81.6%.

Specificity, TN/(TN + FP) = 98.8%.

Precision or positive predictive value (PPV), TP/(TP + FP) = 96.9%.

Negative predictive value (NPV), TN/(TN + FN) = 92.2%.

ACC, (TP + TN)/(TP + FP + FN + TN) = 93.4%.

The classifier is composed of 134 miRNAs. We next determined the average number of miRNAs, out of the 134, that were detected in the plasma of healthy subjects and patients with OSCC. Using only 0.2 ng total miRNA as starting concentration, 93 and 97 miRNAs were detected on average in healthy subjects and patients, respectively (Table 4). The level of miRNAs in plasma is often lower compared to those in tissue samples, but PanelChip™ was capable of detecting a high number of plasma miRNAs, suggesting that the system is well-suited for the detection of circulating miRNAs.

**Table 4:** Average number of miRNAs, out of 134 from the classifier, detected in the plasma of healthy subjects and patients.

	# Detected
HD	93
OSCC	97

HD, healthy donors.

OSCC, patients with OSCC.

^#^Detected, average number of miRNAs detected in the plasma.

## Discussion

In this study, we introduced a new multi-gene expression profiling technology, PanelChip™ Analysis System, for analyzing miRNA expression pattern. The system performed well analytically in the initial miRQC study. In addition, the system demonstrated that it requires only a minute starting amount of miRNAs for experimentations, between 0.2 to 2.5 ng, as shown in this study (see Table 1). Furthermore, we demonstrated the system’s capability in identifying potential miRNA classifiers for diagnostic use. We were able to discover a miRNA expression pattern, utilizing the combination of the system’s miRSCan™ PanCancer Chips and OSCC classifier, capable of separating healthy subjects and patients with OSCC.

A small number of patients were classified as healthy donors in our analysis, mainly due to the number of patients enrolled in the study. We also did not take into account the demographics and clinicopathological factors of the enrolled patients. It would be of great interest to investigate the ability of the system in the discovery and validation of miRNA classifiers with a larger cohort of patients with OSCC.

In conclusion, we demonstrated in this study that by utilizing a novel technology, PanelChip™ Analysis System, miRNA expression patterns specific to patients with OSCC can be discovered. Furthermore, the use of machine learning tools along with system produced an algorithm capable of predicting an individual’s health status with high accuracy. Judging from its performance in the analysis of miRNA expression, we believe that PanelChip™ Analysis System can expedite the discovery and validation of RNA-specific classifiers in cancer diseases.

## Materials and Methods

### Subjects

Thirty-eight patients with untreated primary OSCC and eighty-four healthy subjects were recruited for the study at Chang Gung Memorial Hospital from August 2015 to March 2017, and written informed consent was obtained from all participants. The study was approved by the Institutional Review Board of Chang Gung Memorial Hospital, Linkou, Taiwan (Reference No. 201601461B0 and 102–3136A3), and all experiments were performed in accordance with relevant guidelines and regulations. Blood samples were collected prior to surgery. Samples were processed into plasma within 30 minutes of the collection by two centrifugation steps; samples were first centrifuged at 1200 × g for 10 minutes and the resulting supernatants were then centrifuged at 12000 × g for 10 min. The supernatant, which is plasma, was divided into aliquots and stored at −80 °C immediately. Prior to miRNA extraction, the plasma samples were evaluated for hemolysis based on the absorbance between 414 nm and 375 nm measured by DeNovix. A hemolysis ratio of A414 nm/A375 nm >2 indicates hemolysis and the sample will be excluded from the study. The eight-four healthy donors were between the ages of 22–35 with no prior history of cancer diseases. Samples were processed in the same manner as the patient samples.

### PanelChip™ Analysis System and the production of miRSCan™ PanCancer Chips 1 & 2

PanelChip™ Analysis System was developed as a molecular diagnostic device designed to detect and analyze the gene expression of multiple biomarkers based on polymerase chain reaction amplification technology. The core component of the system facilitating the multi-gene analysis capability is a 36-mm × 36-mm × 1-mm reaction plate called Q-Chip™, which consists of 2500 nanowells, with each nanowell representing one real-time PCR reaction well^34^; Supplementary Fig. S1). In order to perform multiple assays on one Q-Chip™, a proprietary inkjet printing technology deposits primers or primers/probe precisely and accurately into each well without cross contamination into adjacent wells^34^. Theoretically, Q-Chip™ can contain up to 2500 different assays, or clusters (a cluster represents an assay detecting/analyzing a biomarker). A Q-Chip™ containing multiple clusters is called PanelChip™.

PanelStation™ is a real-time quantitative/digital PCR platform developed specifically to run PanelChip™. The thermal cycling functionality is accomplished by Thermal-Roller-Coaster®, a proprietary technology which consists of six resistive heater blocks with different but constant temperatures. Amplification is achieved by shuttling DNA or cDNA samples between denaturing heater blocks and annealing/extension heater blocks. Each PCR run can accommodate up to 6 samples and controls. The platform utilizes a white-light LED optics system with up to 4 different filter block formats to achieve illumination for samples with FAM^TM^, VIC®, ROX^TM^, and Cy5^TM^ dye.

miRSCan™ PanCancer Chips 1 & 2 are PanelChips™ with selected miRNA targets based on miDatabase™ (Yang, K. C. *et al*. in submission). The candidates were selected, from published literatures, based on their implications as cancer biomarkers and their presence in bodily fluids. miRSCan™ PanCancer Chips 1 & 2 contain a total of 164 biomarkers and 7 controls, where all biomarkers and controls have 9 repeats within the same chip (see Supplementary Fig. S1 and Table S4). Primer pairs for each miRNA biomarker was deposited into the reaction nanowell, in which the amount deposited will give a final qPCR reaction concentration of 0.25 µM. Primers were designed using miPrimer™ methodology^40^. All oligonucleotides were purchased from IDT.

### Plasma preparation

Blood was collected from a cubital vein into a K2EDTA BD Vacutainer tube through a 21 G needle. Plasma was isolated within 30 minutes of the blood draw by centrifugation at 1200 × g at room temperature for 10 min in a swing bucket rotor. 2 ml supernatant was transferred to a new, labeled 15 ml tube without disturbing the buffy coat. Plasma samples with a hemoglobin concentration of ≦50 mg/dL were then centrifuged at 12,000 × g at room temperature for 10 min. Supernatant was transferred to a new, labeled 15 ml tube without disturbing the sediment at the bottom of the tube. The plasma sample was then divided into 600 µl aliquots and stored at −80 °C immediately.

### miRNA extraction

For clinical samples, miRNA was isolated from 600 µl of plasma with Macherey-Nagel’s NucleopSpin® miRNA Plasma kit (740981.250) following the manufacturer’s protocol. Plasma miRNAs were eluted in 30 μL nuclease-free water. The concentration and the quality of the extracted miRNAs were quantified using ThermoFisher’s Qubit® microRNA Assay Kit (Q32880) and analyzed using Agilent’s BioAnalyzer® Small RNA Kit (5067-1548), respectively. For miRQC experiments, plasma miRNAs from three healthy donors were pooled together for further experiments. Synthetic hsa-let-7a-5p, hsa-let-7b-5p and hsa-let-7c-5p miRNAs (IDT) were spiked into MS2-phage RNA without endogenous let-7 miRNAs (Sigma-Aldrich, #Roche-10165948001) to a final concentration of 5 × 106 copies/µg RNA (for samples 9–11).

### RNA samples for miRQC analysis

Universal Human miRNA reference RNA (miRQC A for samples 1–2; Agilent Technologies, #750700) and human brain total RNA (miRQC B for samples 3–4; Life Technologies, AM7960) were purchased from Agilent and Life Technologies, respectively. 300 ng of RNA from miRQC A (75%) and 100 ng of RNA from miRQC B (25%) were mixed together to obtain miRQC C for samples 5–6. 100 ng of RNA from miRQC A (25%) and 300 ng of RNA from miRQC B (75%) were mixed together to obtain miRQC D for samples 7–8. Serial dilutions of synthetic miR-10a-5p miRNA (IDT) were spiked into 20 ng of Universal Human miRNA reference RNA (without endogenous miR-10a-5p) to a final concentration ranging from 1 × 1011 copies/ng RNA to 1 × 105 copies/ng RNA over 6 logs.

### cDNA synthesis

For miRQC samples 1–8, 400 ng of total RNA were used to synthesize cDNA in 20 µl reverse transcription reactions. For miRQC samples 9–11, 200 ng of MS2 phage RNA with spiked-in let-7 miRNAs were used to synthesize cDNA in 20 µl reverse transcription reactions. For miRQC samples 12–15, 2 ng of total miRNAs were used to synthesize cDNA in 20 µl reverse transcription reactions. For miRQC samples 16–22, 20 ng of RNA with spiked-in miR-10a-5p miRNA were used to synthesize cDNA in 20 µl reverse transcription reactions. 2 ng of total miRNAs from patient and healthy donor samples were used to synthesize cDNA in 20 µl reverse transcription reactions. The reverse transcription step was performed as follows: Poly-A tail was added to the miRNA population using Poly-A polymerase, followed by cDNA synthesis with QuarkBio’s microRNA Reverse Transcription kit (Quark Biosciences, Inc.).

### qPCR utilizing PanelChip™ Analysis System and miRSCan™ PanCancer Chips 1 & 2

For miRQC studies, 2.5 μl of the cDNA template (2.5 ng cDNA) for samples 1–11, 2 μl of the cDNA template (0.2 ng cDNA) for samples 12–15, and 2 μl of the cDNA template (2 ng cDNA) for samples 16–22 were added to the qPCR mixture containing 30 μl of 2 × QuarkBio qPCR master mix (Quark Biosciences, Inc.). For samples from patients and healthy donors, 0.2 ng cDNA was added to the qPCR mixture containing 30 μl of 2 × QuarkBio qPCR master mix (Quark Biosciences, Inc.). Nuclease-free water was added to the mixture to obtain a final volume of 60 μl. The master mix was mixed thoroughly, and briefly spun down to collect the liquid at the bottom. To apply the qPCR mixture to be analyzed on to chips 1 & 2, 60 μl of the mixture was dispensed using a Pipetman along the edge of the chips. The qPCR mixture should cover the entire length of the 50 wells along the edge of the chip. 50 μl of the qPCR mixture is then applied across the entire surface of the chip via a scraping motion with a glass slide, resulting in 20 nl of master mix per reaction well. The chip is then submerged, into a tray containing mineral oil, with reaction wells facing the bottom of the tray. Each tray is then placed into PanelStation™ for amplification of the templates and signal detection. qPCR was subsequently performed according to the following cycling program: 95 °C for 36 seconds and 60 °C for 72 seconds for 40 cycles.

### Data preprocessing for miRQC analysis and OSCC classifier

Data from the miRQC study was preprocessed based on the samples listed in Supplementary Table S2. The miRQC analyses listed in Tables 1–2 were referenced to the original miRQC study, which was conducted by Mestdagh *et al*.^37^. All miRQC profiles were filtered based on the cutoff number listed in Table 1. miRQC A-D were further normalized using the five factors, including hsa-RNU6B, hsa-RNU43, hsa-18s rRNA, miRNA reverse transcription (RT) and qPCR controls.

Data obtained from at least two biological repeat runs of miRSCan™ PanCancer Chips 1 & 2 were preprocessed and modeled on the 38 OSCC patients and 84 healthy donors. A total of 357 miRSCan™ PanCancer profiles were normalized using the miRNA RT and qPCR controls. The miRNA RT control and qPCR control were spiked in during reverse transcription and qPCR, respectively, as process controls^40^. After averaging the replicates, 122 normalized miRSCan™ PanCancer profiles remained for further analysis. For subsequent data preprocessing, miRNAs without amplification signals across all profiles were removed, resulting in 134 miRNAs; the missing miRNA values for the individual profile were replaced with the maximum ΔCq of the entire profile; each profile was standardized by zero mean and unit variance.

miRSCan™ PanCancer profiles were categorized into two classes: OSCC and HD. The ΔCq values of 134 selected miRNAs were used as features. With these features, the LIBSVM in R package e1071 (version 1.6-8) was used to build an OSCC classifier^45^. 10-fold cross validation was used to evaluate the performance of the OSCC classifier.

## Electronic supplementary material

Supplementary Information
