# Supplementary material for: A Novel Multi-Gene Detection Platform for the Analysis of miRNA Expression
Source: Sci Rep. 2018 Jul 16;8:10684. doi: 10.1038/s41598-018-29146-7 (PMC6048151; doi:10.1038/s41598-018-29146-7)
Supplement: Supplementary file 1 — Supplementary Information [file 41598_2018_29146_MOESM1_ESM.docx]

# A Novel Multi-Gene Detection Platform for the Analysis of miRNA Expression

Chia-Hsun Hsieh^1¶^, Wei-Ming Chen^2¶^, Yi-Shan Hsieh^2^, Ya-Chun Fan^2^, Pok Eric Yang^2^, Shih-Ting Kang^2^*, Chun-Ta Liao^3^*

^1^Division of Hematology-Oncology, Department of Internal Medicine, Chang Gung Memorial Hospital, Taoyuan, Taiwan

^2^Department of Application Development, Quark Biosciences, Inc., Zhubei City, Hsinchu, Taiwan

^3^ Department of Otorhinolaryngology, Head & Neck Surgery, Linkou Chang Gung Memorial Hospital and Chang Gung University, Taoyuan, Taiwan

^¶^ These authors contributed equally to this work.

* Corresponding authors

Email: [liaoct@cgmh.org.tw](mailto:liaoct@cgmh.org.tw) (CTL), [benniekang@quarkbiosciences.com](mailto:benniekang@quarkbiosciences.com) (STK)

# Supplementary information

**Supplementary Figure S1. Schematic drawing of a PanelChip™ with 96 clusters, with each clusters having 9 replicates.** Using proprietary arraying technology, miRNA primers were printed directly into each nanowell. Each cluster of 9 nanowell replicates represents a miRNA assay or a control assay.


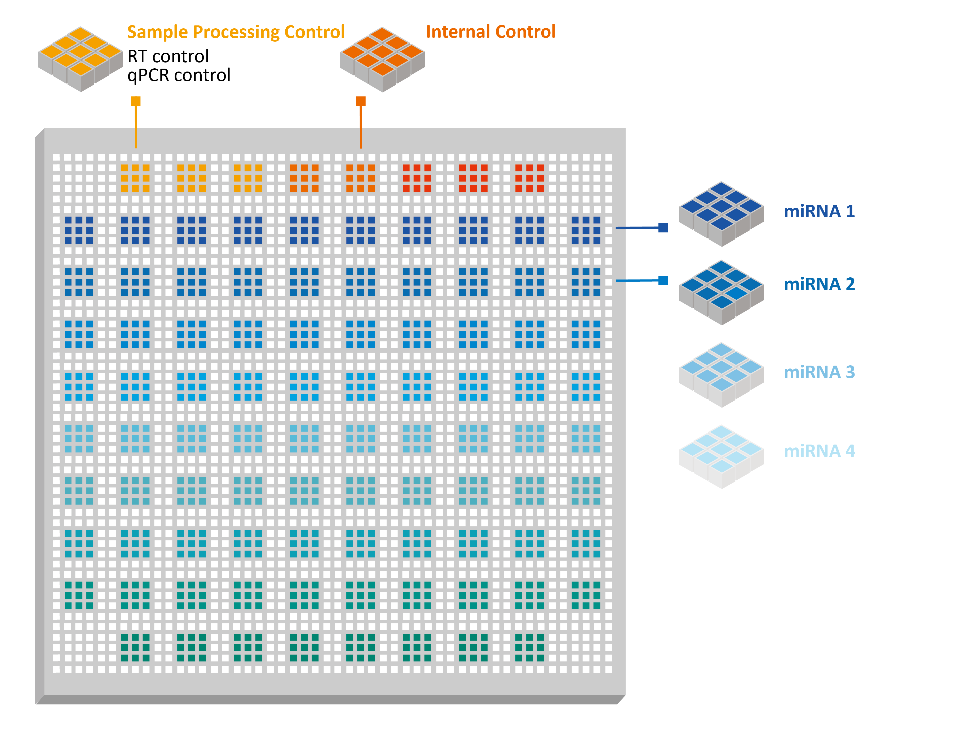


**Supplementary Table S1. PCR efficiency of miRNA assays on miRSCan PanCancer Chip 1&2.**

| **miRNA name** | **PCR efficiency** |
| --- | --- |
| hsa-let-7a-5p | 89% |
| hsa-let-7b-5p | 89% |
| hsa-let-7c-5p | 88% |
| hsa-let-7d-3p | 94% |
| hsa-let-7f-5p | 89% |
| hsa-let-7g-5p | 94% |
| hsa-miR-100-5p | 99% |
| hsa-miR-101-3p | 96% |
| hsa-miR-106a-5p | 92% |
| hsa-miR-106b-5p | 89% |
| hsa-miR-10a-5p | 93% |
| hsa-miR-10b-5p | 92% |
| hsa-miR-1225-3p | 97% |
| hsa-miR-1228-5p | 93% |
| hsa-miR-1254 | 99% |
| hsa-miR-125b-5p | 94% |
| hsa-miR-127-3p | 100% |
| hsa-miR-128-3p | 96% |
| hsa-miR-1290 | 94% |
| hsa-miR-130a-3p | 92% |
| hsa-miR-130b-3p | 93% |
| hsa-miR-133a-3p | 99% |
| hsa-miR-134-5p | 98% |
| hsa-miR-135a-5p | 91% |
| hsa-miR-135b-5p | 93% |
| hsa-miR-1-3p | 97% |
| hsa-miR-140-5p | 94% |
| hsa-miR-141-3p | 90% |
| hsa-miR-142-3p | 95% |
| hsa-miR-143-3p | 93% |
| hsa-miR-145-5p | 98% |
| hsa-miR-146a-5p | 95% |
| hsa-miR-146b-5p | 97% |
| hsa-miR-151a-3p | 95% |
| hsa-miR-152-3p | 94% |
| hsa-miR-155-5p | 93% |
| hsa-miR-15b-5p | 91% |
| hsa-miR-16-5p | 95% |
| hsa-miR-17-3p | 91% |
| hsa-miR-17-5p | 91% |
| hsa-miR-181a-5p | 92% |
| hsa-miR-181b-5p | 93% |
| hsa-miR-182-5p | 96% |
| hsa-miR-183-5p | 94% |
| hsa-miR-18a-5p | 94% |
| hsa-miR-18b-5p | 95% |
| hsa-miR-191-5p | 98% |
| hsa-miR-192-5p | 99% |
| hsa-miR-193a-3p | 96% |
| hsa-miR-195-5p | 93% |
| hsa-miR-196a-5p | 92% |
| hsa-miR-196b-5p | 90% |
| hsa-miR-1972 | 98% |
| hsa-miR-197-3p | 96% |
| hsa-miR-198 | 97% |
| hsa-miR-199a-5p | 97% |
| hsa-miR-19a-3p | 96% |
| hsa-miR-19b-3p | 91% |
| hsa-miR-200a-3p | 98% |
| hsa-miR-200b-3p | 96% |
| hsa-miR-200c-3p | 95% |
| hsa-miR-202-3p | 95% |
| hsa-miR-203a-3p | 98% |
| hsa-miR-206 | 94% |
| hsa-miR-20a-5p | 92% |
| hsa-miR-20b-5p | 96% |
| hsa-miR-210-3p | 99% |
| hsa-miR-2114-3p | 96% |
| hsa-miR-214-3p | 96% |
| hsa-miR-215-5p | 95% |
| hsa-miR-21-5p | 93% |
| hsa-miR-216a-5p | 94% |
| hsa-miR-221-3p | 95% |
| hsa-miR-222-3p | 95% |
| hsa-miR-223-3p | 97% |
| hsa-miR-22-3p | 94% |
| hsa-miR-224-5p | 96% |
| hsa-miR-23a-3p | 98% |
| hsa-miR-24-3p | 93% |
| hsa-miR-26a-5p | 96% |
| hsa-miR-27a-3p | 96% |
| hsa-miR-28-3p | 94% |
| hsa-miR-299-5p | 96% |
| hsa-miR-29a-3p | 97% |
| hsa-miR-29a-5p | 95% |
| hsa-miR-29c-3p | 89% |
| hsa-miR-302d-3p | 95% |
| hsa-miR-30a-5p | 93% |
| hsa-miR-30b-5p | 96% |
| hsa-miR-30c-5p | 99% |
| hsa-miR-30d-5p | 94% |
| hsa-miR-30e-5p | 96% |
| hsa-miR-31-5p | 96% |
| hsa-miR-326 | 98% |
| hsa-miR-335-5p | 94% |
| hsa-miR-338-5p | 95% |
| hsa-miR-34a-5p | 90% |
| hsa-miR-361-5p | 94% |
| hsa-miR-372-3p | 97% |
| hsa-miR-373-3p | 97% |
| hsa-miR-375 | 97% |
| hsa-miR-376c-3p | 89% |
| hsa-miR-378a-5p | 96% |
| hsa-miR-382-5p | 93% |
| hsa-miR-409-3p | 96% |
| hsa-miR-411-5p | 100% |
| hsa-miR-423-3p | 100% |
| hsa-miR-423-5p | 99% |
| hsa-miR-425-3p | 96% |
| hsa-miR-425-5p | 98% |
| hsa-miR-451a | 97% |
| hsa-miR-452-3p | 94% |
| hsa-miR-483-5p | 96% |
| hsa-miR-484 | 99% |
| hsa-miR-486-5p | 96% |
| hsa-miR-499a-5p | 94% |
| hsa-miR-500a-5p | 96% |
| hsa-miR-518a-5p | 96% |
| hsa-miR-520b | 94% |
| hsa-miR-574-3p | 97% |
| hsa-miR-574-5p | 98% |
| hsa-miR-579-3p | 96% |
| hsa-miR-589-5p | 98% |
| hsa-miR-593-5p | 98% |
| hsa-miR-596 | 92% |
| hsa-miR-601 | 96% |
| hsa-miR-625-5p | 98% |
| hsa-miR-652-3p | 95% |
| hsa-miR-660-5p | 97% |
| hsa-miR-663a | 99% |
| hsa-miR-718 | 92% |
| hsa-miR-7-5p | 96% |
| hsa-miR-760 | 96% |
| hsa-miR-885-5p | 98% |
| hsa-miR-93-5p | 91% |
| hsa-miR-940 | 96% |
| hsa-miR-95-3p | 97% |
| hsa-miR-9-5p | 95% |
| hsa-miR-99b-5p | 96% |

**Supplementary Table S2. Sets of standard samples used for miRQC analysis.**

| **Sample number** | **Sample name** | **Spike-in** | **Spike-in concentration** |
| --- | --- | --- | --- |
| 1 | miRQC A | - | - |
| 2 | miRQC A | - | - |
| 3 | miRQC B | - | - |
| 4 | miRQC B | - | - |
| 5 | miRQC C | - | - |
| 6 | miRQC C | - | - |
| 7 | miRQC D | - | - |
| 8 | miRQC D | - | - |
| 9 | MS2 phage | let-7a-5p | 5e6 |
| 10 | MS2 phage | let-7b-5p | 5e6 |
| 11 | MS2 phage | let-7c | 5e6 |
| 12 | Plasma | - |  |
| 13 | Plasma | - |  |
| 14 | Plasma | - |  |
| 15 | Plasma | - |  |
| 16 | miRQC A | miR-10a-5p | 1e11 |
| 17 | miRQC A | miR-10a-5p | 1e10 |
| 18 | miRQC A | miR-10a-5p | 1e9 |
| 19 | miRQC A | miR-10a-5p | 1e8 |
| 20 | miRQC A | miR-10a-5p | 1e7 |
| 21 | miRQC A | miR-10a-5p | 1e6 |
| 22 | miRQC A | miR-10a-5p | 1e5 |

miRQC A, 100% Universal Human miRNA Reference RNA.

miRQC B, 100% human brain RNA.

miRQC C, 75% miRQC A and 25% miRQC B.

miRQC D, 25% miRQC A and 75% miRQC B.

Spike-in, synthetic RNA oligonucleotides added to the sample.

Spike-in concentration, the amount of spike-in added to the sample.

MS Phage, MS Phage RNA.

Plasma, total small RNAs extracted from plasma.

**Supplementary Table S3. Demographic and clinical information of patients with OSCC.**

| **Patient ID** | **Age** | **Gender** | **Pathologic Tumor stage** |
| --- | --- | --- | --- |
| OSCC001 | 50 | Male | T2N0M0 |
| OSCC002 | 49 | Male | T2N0M0 |
| OSCC003 | 50 | Male | T2N2bM0 |
| OSCC004 | 50 | Male | T2N0M0 |
| OSCC006 | 58 | Male | T4N0M0 |
| OSCC007 | 36 | Male | T4N0M0 |
| OSCC009 | 56 | Male | T4N2cM0 |
| OSCC010 | 47 | Male | T4N0M0 |
| OSCC011 | 65 | Male | T2N0 M0 |
| OSCC013 | 40 | Male | T4aN0M0 |
| OSCC014 | 76 | Male | T4aN2bM0 |
| OSCC015 | 43 | Male | T4aN2CM0 |
| OSCC016 | 35 | Male | T4a N2bM0 |
| OSCC017 | 65 | Male | T2N1M0 |
| OSCC018 | 46 | Male | T4aN0M0 |
| OSCC020 | 39 | Male | T2N0M0 |
| OSCC021 | 54 | Male | T3N1M0 |
| OSCC023 | 56 | Male | T4aN0M0 |
| OSCC024 | 56 | Male | T4aN1M0 |
| OSCC025 | 71 | Female | T1N0M0 |
| OSCC027 | 50 | Male | T3N0M0 |
| OSCC028 | 67 | Male | T2N0M0 |
| OSCC029 | 53 | Male | T2N0M0 |
| OSCC030 | 56 | Male | T4aN0M0 |
| OSCC031 | 43 | Male | T2N1M0 |
| OSCC032 | 53 | Male | T2N0M0 |
| OSCC033 | 58 | Male | T2N0M0 |
| OSCC034 | 59 | Male | T3N1M0 |
| OSCC035 | 37 | Female | T2N0M0 |
| OSCC037 | 54 | Male | T4bN0M0 |
| OSCC038 | 56 | Male | T2N0M0 |
| OSCC043 | 54 | Male | T2N2bM0 |
| OSCC044 | 46 | Male | T3N2bM0 |
| OSCC045 | 40 | Male | T2N0M0 |
| OSCC046 | 46 | Male | T3N0M0 |
| OSCC047 | 62 | Male | T3N2bM0 |
| OSCC048 | 49 | Male | T4aN2cM0 |
| OSCC051 | 62 | Male | T2N2bM0 |

T, size or direct extent of primary tumor

N, degree of spread to regional lymph nodes

M, presence of distant metastasis

**Supplementary Table S4. Controls and miRNAs on miRSCan™ PanCancer Chip 1 & 2.**

| **Chip** | **miRNA name** | **Annotation** | **OSCC study** |
| --- | --- | --- | --- |
| 1 | cel-miR-39 | Control for RNA extraction | N |
| 1 | hsa-RNU6B | Endogeneous control | N |
| 1 | hsa-RNU43 | Endogeneous control | N |
| 1 | hsa-18srRNA | Endogeneous control | N |
| 1 | RT control | Control for cDNA synthesis | N |
| 1 | qPCR control | Control for qPCR reaction | N |
| 1 | hsa-18srRNA-ITS | Control for gDNA contamination | N |
| 1 | hsa-let-7a-5p | miRNA biomarker | Y |
| 1 | hsa-miR-2114-3p | miRNA biomarker | Y |
| 1 | hsa-miR-20a-5p | miRNA biomarker | Y |
| 1 | hsa-miR-222-3p | miRNA biomarker | Y |
| 1 | hsa-miR-143-3p | miRNA biomarker | Y |
| 1 | hsa-miR-17-3p | miRNA biomarker | Y |
| 1 | hsa-miR-181a-5p | miRNA biomarker | Y |
| 1 | hsa-miR-103a-3p | miRNA biomarker | Y |
| 1 | hsa-miR-202-3p | miRNA biomarker | Y |
| 1 | hsa-let-7d-3p | miRNA biomarker | Y |
| 1 | hsa-miR-101-3p | miRNA biomarker | Y |
| 1 | hsa-miR-122-5p | miRNA biomarker | Y |
| 1 | hsa-miR-1254 | miRNA biomarker | Y |
| 1 | hsa-miR-125b-5p | miRNA biomarker | Y |
| 1 | hsa-miR-126-3p | miRNA biomarker | Y |
| 1 | hsa-miR-1290 | miRNA biomarker | Y |
| 1 | hsa-miR-142-3p | miRNA biomarker | Y |
| 1 | hsa-miR-145-5p | miRNA biomarker | Y |
| 1 | hsa-miR-146a-5p | miRNA biomarker | Y |
| 1 | hsa-miR-150-5p | miRNA biomarker | Y |
| 1 | hsa-miR-151a-3p | miRNA biomarker | Y |
| 1 | hsa-miR-152-3p | miRNA biomarker | Y |
| 1 | hsa-miR-155-5p | miRNA biomarker | Y |
| 1 | hsa-miR-15a-5p | miRNA biomarker | Y |
| 1 | hsa-miR-15b-5p | miRNA biomarker | Y |
| 1 | hsa-miR-1225-3p | miRNA biomarker | Y |
| 1 | hsa-miR-181b-5p | miRNA biomarker | Y |
| 1 | hsa-miR-182-5p | miRNA biomarker | Y |
| 1 | hsa-miR-183-5p | miRNA biomarker | Y |
| 1 | hsa-miR-193a-3p | miRNA biomarker | Y |
| 1 | hsa-miR-1972 | miRNA biomarker | Y |
| 1 | hsa-miR-197-3p | miRNA biomarker | Y |
| 1 | hsa-miR-199a-5p | miRNA biomarker | Y |
| 1 | hsa-miR-19a-3p | miRNA biomarker | Y |
| 1 | hsa-miR-19b-3p | miRNA biomarker | Y |
| 1 | hsa-miR-203a-3p | miRNA biomarker | Y |
| 1 | hsa-miR-205-5p | miRNA biomarker | Y |
| 1 | hsa-miR-206 | miRNA biomarker | Y |
| 1 | hsa-miR-214-3p | miRNA biomarker | Y |
| 1 | hsa-miR-215-5p | miRNA biomarker | Y |
| 1 | hsa-miR-21-5p | miRNA biomarker | Y |
| 1 | hsa-miR-16-5p | miRNA biomarker | Y |
| 1 | hsa-miR-191-5p | miRNA biomarker | Y |
| 1 | hsa-miR-22-3p | miRNA biomarker | Y |
| 1 | hsa-miR-224-5p | miRNA biomarker | Y |
| 1 | hsa-miR-24-3p | miRNA biomarker | Y |
| 1 | hsa-miR-26a-5p | miRNA biomarker | Y |
| 1 | hsa-miR-140-5p | miRNA biomarker | Y |
| 1 | hsa-miR-28-3p | miRNA biomarker | Y |
| 1 | hsa-miR-299-5p | miRNA biomarker | Y |
| 1 | hsa-miR-29a-5p | miRNA biomarker | Y |
| 1 | hsa-miR-23a-3p | miRNA biomarker | Y |
| 1 | hsa-miR-423-3p | miRNA biomarker | Y |
| 1 | hsa-miR-198 | miRNA biomarker | Y |
| 1 | hsa-miR-31-5p | miRNA biomarker | Y |
| 1 | hsa-miR-129-5p | miRNA biomarker | N |
| 1 | hsa-miR-326 | miRNA biomarker | Y |
| 1 | hsa-miR-335-5p | miRNA biomarker | Y |
| 1 | hsa-miR-338-5p | miRNA biomarker | Y |
| 1 | hsa-miR-361-5p | miRNA biomarker | Y |
| 1 | hsa-miR-372-3p | miRNA biomarker | Y |
| 1 | hsa-miR-451a | miRNA biomarker | Y |
| 1 | hsa-miR-423-5p | miRNA biomarker | Y |
| 1 | hsa-miR-378a-5p | miRNA biomarker | Y |
| 1 | hsa-miR-382-5p | miRNA biomarker | Y |
| 1 | hsa-miR-409-3p | miRNA biomarker | Y |
| 1 | hsa-miR-425-3p | miRNA biomarker | Y |
| 1 | hsa-miR-452-3p | miRNA biomarker | N |
| 1 | hsa-miR-483-5p | miRNA biomarker | Y |
| 1 | hsa-miR-484 | miRNA biomarker | Y |
| 1 | hsa-miR-499a-5p | miRNA biomarker | Y |
| 1 | hsa-miR-93-5p | miRNA biomarker | Y |
| 1 | hsa-miR-425-5p | miRNA biomarker | Y |
| 1 | hsa-miR-574-3p | miRNA biomarker | Y |
| 1 | hsa-miR-574-5p | miRNA biomarker | Y |
| 1 | hsa-miR-579-3p | miRNA biomarker | Y |
| 1 | hsa-miR-589-5p | miRNA biomarker | Y |
| 1 | hsa-miR-593-5p | miRNA biomarker | Y |
| 1 | hsa-miR-596 | miRNA biomarker | Y |
| 1 | hsa-miR-601 | miRNA biomarker | Y |
| 1 | hsa-miR-216a-5p | miRNA biomarker | Y |
| 1 | hsa-miR-940 | miRNA biomarker | Y |
| 1 | hsa-miR-34a-5p | miRNA biomarker | Y |
| 1 | hsa-miR-30b-5p | miRNA biomarker | Y |
| 1 | hsa-miR-373-3p | miRNA biomarker | Y |
| 1 | hsa-miR-375 | miRNA biomarker | Y |
| 1 | hsa-miR-500a-5p | miRNA biomarker | Y |
| 1 | hsa-miR-144-3p | miRNA biomarker | N |
| 1 | hsa-miR-1228-5p | miRNA biomarker | Y |
| 2 | hsa-let-7b-5p | miRNA biomarker | Y |
| 2 | hsa-let-7c-5p | miRNA biomarker | Y |
| 2 | hsa-let-7d-5p | miRNA biomarker | Y |
| 2 | hsa-let-7f-5p | miRNA biomarker | Y |
| 2 | hsa-let-7g-5p | miRNA biomarker | Y |
| 2 | hsa-miR-100-5p | miRNA biomarker | Y |
| 2 | hsa-miR-10a-5p | miRNA biomarker | Y |
| 2 | hsa-miR-10b-5p | miRNA biomarker | Y |
| 2 | hsa-miR-130a-3p | miRNA biomarker | Y |
| 2 | hsa-miR-130b-3p | miRNA biomarker | Y |
| 2 | hsa-miR-133a-3p | miRNA biomarker | Y |
| 2 | hsa-miR-133b-3p | miRNA biomarker | N |
| 2 | hsa-miR-134-5p | miRNA biomarker | Y |
| 2 | hsa-miR-135b-5p | miRNA biomarker | N |
| 2 | hsa-miR-106a-5p | miRNA biomarker | Y |
| 2 | hsa-miR-141-3p | miRNA biomarker | Y |
| 2 | hsa-miR-146b-5p | miRNA biomarker | Y |
| 2 | hsa-miR-208a-3p | miRNA biomarker | N |
| 2 | hsa-miR-193a-5p | miRNA biomarker | N |
| 2 | hsa-miR-17-5p | miRNA biomarker | Y |
| 2 | hsa-miR-18a-5p | miRNA biomarker | Y |
| 2 | hsa-miR-18b-5p | miRNA biomarker | Y |
| 2 | hsa-miR-192-5p | miRNA biomarker | Y |
| 2 | hsa-miR-195-5p | miRNA biomarker | Y |
| 2 | hsa-miR-196a-5p | miRNA biomarker | N |
| 2 | hsa-miR-196b-5p | miRNA biomarker | N |
| 2 | hsa-miR-200a-3p | miRNA biomarker | Y |
| 2 | hsa-miR-200b-3p | miRNA biomarker | Y |
| 2 | hsa-miR-200c-3p | miRNA biomarker | N |
| 2 | hsa-miR-20b-5p | miRNA biomarker | Y |
| 2 | hsa-miR-210-3p | miRNA biomarker | Y |
| 2 | hsa-miR-193b-5p | miRNA biomarker | N |
| 2 | hsa-miR-221-3p | miRNA biomarker | Y |
| 2 | hsa-miR-27a-3p | miRNA biomarker | Y |
| 2 | hsa-miR-29a-3p | miRNA biomarker | Y |
| 2 | hsa-miR-29c-3p | miRNA biomarker | Y |
| 2 | hsa-miR-30a-5p | miRNA biomarker | Y |
| 2 | hsa-miR-30c-5p | miRNA biomarker | Y |
| 2 | hsa-miR-30d-5p | miRNA biomarker | Y |
| 2 | hsa-miR-30e-5p | miRNA biomarker | Y |
| 2 | hsa-miR-376c-3p | miRNA biomarker | Y |
| 2 | hsa-miR-411-5p | miRNA biomarker | Y |
| 2 | hsa-miR-520b | miRNA biomarker | Y |
| 2 | hsa-miR-486-5p | miRNA biomarker | Y |
| 2 | hsa-miR-223-3p | miRNA biomarker | Y |
| 2 | hsa-miR-1-3p | miRNA biomarker | Y |
| 2 | hsa-miR-625-5p | miRNA biomarker | Y |
| 2 | hsa-miR-652-3p | miRNA biomarker | Y |
| 2 | hsa-miR-660-5p | miRNA biomarker | Y |
| 2 | hsa-miR-663a | miRNA biomarker | Y |
| 2 | hsa-miR-718 | miRNA biomarker | Y |
| 2 | hsa-miR-7-5p | miRNA biomarker | Y |
| 2 | hsa-miR-760 | miRNA biomarker | Y |
| 2 | hsa-miR-885-5p | miRNA biomarker | Y |
| 2 | hsa-miR-92a-3p | miRNA biomarker | Y |
| 2 | hsa-miR-95-3p | miRNA biomarker | Y |
| 2 | hsa-miR-208b-3p | miRNA biomarker | N |
| 2 | hsa-miR-29b-3p | miRNA biomarker | N |
| 2 | hsa-miR-302d-3p | miRNA biomarker | N |
| 2 | hsa-miR-106b-5p | miRNA biomarker | N |
| 2 | hsa-miR-127-3p | miRNA biomarker | N |
| 2 | hsa-miR-450a-5p | miRNA biomarker | N |
| 2 | hsa-miR-518a-5p | miRNA biomarker | N |
| 2 | hsa-miR-9-5p | miRNA biomarker | N |
| 2 | hsa-miR-99b-5p | miRNA biomarker | N |
| 2 | hsa-miR-128-3p | miRNA biomarker | N |
| 2 | hsa-miR-135a-5p | miRNA biomarker | N |
| 2 | hsa-miR-331-3p | miRNA biomarker | N |
| 2 | hsa-miR-320a | miRNA biomarker | N |
| 2 | hsa-miR-199a-3p | miRNA biomarker | N |
| 2 | hsa-miR-330-5p | miRNA biomarker | N |
| 2 | hsa-miR-1248 | miRNA biomarker | N |
| 2 | hsa-miR-429 | miRNA biomarker | N |
| 2 | hsa-miR-3605-3p | miRNA biomarker | N |
| 2 | hsa-miR-4306 | miRNA biomarker | N |
| 2 | hsa-miR-302a-3p | miRNA biomarker | N |
| 2 | hsa-miR-27b-3p | miRNA biomarker | N |
| 2 | hsa-miR-30e-3p | miRNA biomarker | N |

Chip, miRSCan™ PanCancer 1 or 2.

OSCC study, miRNA used in the analysis of clinical samples.

Y and N, represents miRNAs that have been or have not been, respectively, in classifying subjects.
